# Comparison of Epidemiological Methods for Estimation of Hepatitis B Incidence and Residual Risk for Blood Donors in Southern Brazil

**DOI:** 10.4061/2011/985383

**Published:** 2011-05-10

**Authors:** Emil Kupek, Andrea Petry

**Affiliations:** ^1^Departamento de Saúde Pública/CCS, Universidade Federal de Santa Catarina, 88040-900 Florianopolis, SC, Brazil; ^2^Hematology and Hemotherapy Center of Santa Catarina (HEMOSC), Florianopolis, SC, Brazil

## Abstract

*Background and Objective*. The objective of this work was to compare three methods for estimating hepatitis B virus (HBV) incidence and residual risk. *Methods*. Computerized blood donor records in southern Brazil were examined for the period 2004–2006. The methods for estimating HBV incidence included stand-alone HBsAg, HBsAg yield method, and an extension of the latter which added recent anti-HBc seroconversions as incident HBV cases. *Results*. HBV incidences for the above methods were 9.91, 20.09, and 22.93 per 100000 repeat donors, respectively. In the same order, corresponding residual risks were 1 : 62482, 1 : 30821, and 1 : 47559, respectively. First-time donors had 52 higher HBV incidence compared to repeat donors. *Conclusion*. Although the three methods compared produced overlapping 95% confidence intervals, their variation was considerably lower for the method which included recent anti-HBc seroconversions. First-time donors are primary cause for concern regarding HBV transmission via blood transfusion in southern Brazil.

## 1. Introduction

There is a consensus that infection caused by hepatitis B virus (HBV) is a worldwide public health concern of high priority, given over 2 billion people who have already been infected and about 350 million of them living with a chronic infection [1]. About a million deaths per year are attributed to this infection and its complications, primarily liver cancer and cirrhosis [1, 2]. 

Three quarters of the world population live in high-prevalence areas for HBV [2]. In developing countries with endemic regions for this infection, such as the Southeast of Asia, China, Africa, many Middle East countries, Pacific islands, and the Amazon region, serologic surveys show that majority of the population has been infected and that 8–15% are chronic HBV carriers. Because 5–12% of the women of childbearing age have been infected with HBV, the risk of perinatal transmission reaches the level of 70–90%, resulting in many newborns infected at birth. In addition, many children are infected during childhood through direct contact with infected blood, ulcerative wounds, or saliva. Approximately one quarter of the children infected with HBV until five years of age become chronic carriers of the virus. On the other hand, adolescent and adult populations acquire HBV through sexual contacts and have between 1% and 5% risk of becoming chronic carriers [3].

Such a high disease burden is even more absurd in the light of the fact that HBV infection is preventable by affordable vaccine (about U$10 per person for the standard vaccine schedule). Among developing countries, Brazil has recently started its own production of a DNA-recombinant hepatitis B vaccine whose effectiveness has been confirmed in various studies [4–6]. Other important measures to prevent HBV transmission include screening the blood donors and pregnant women. 

In Brazil, at least 15% of the population has been infected with HBV, and about 1% of them remain as chronic carriers [7]. As a consequence, the residual risk for not detecting the virus through serological tests for blood donor screening is also high compared to the developed countries. Routine blood screening for HBV in Brazil includes HBsAg (HBV surface antigen) and anti-HBc (antigen to HBV core antibody). The residual risk is the product of the infectious window period and the virus incidence in blood donor population [8, 9]. The latter depends primarily on the effectiveness of predonation interview in eliminating donor candidates with risky sexual behavior. However, other important factors for HBV prevalence in the general population such as the risk of perinatal transmission and vaccine coverage are not within the scope of blood donor selection procedures. 

Despite a significant reduction in residual risk for HBV in southern Brazil in the past two decades [10–13], it is still much higher than in the developed countries. In addition, the risk reduction was not linear in time, showing temporary peaks whose significance has not yet been elucidated [11]. Although nucleid acid testing (NAT) for human immunodeficiency virus (HIV) and hepatitis C has recently started as a pilot scheme in some Brazilian blood banks, it seems unlikely that it is going to be extended to HBV. Therefore, a significant reduction in infectious window period due to the NAT is not a viable option for reducing the residual risk for HBV in Brazil in near future. It is thus necessary to implement alternative methods to estimate the virus incidence in blood donors. 

The most frequently used method to estimate HBV incidence in blood donors is based on the serologic marker HbsAg alone, with adjustment for approximately one quarter of the population with primary antibody response [8, 9]. Although widely applied in various countries [14], including in Brazil1 [1, 15], this method is restricted to repeat donors and depends on the duration of interdonation interval which is pretty variable between the blood banks. In addition, this method requires a large number of repeat donors to obtain reasonably accurate estimates [16].

Another way to estimate HBV incidence in blood donors is by testing for both immunoglobulin G (IgG) and M (IgM) for anti-HBc to distinguish between an old and a recent infection. Thus a recent (<6 moths) infection is diagnosed if either IgM anti-HBc or HBsAg is positive. However, these are not serological tests used for routine screening as only total anti-HBc is used for this purpose worldwide. The same restriction is applied to yet another method to estimate HBV incidence in blood donors based on anti-HBc positive test result and any of the following: a positive test result for anti-HBe (antigen to HBV antibody “e”), anti-HBc IgM, DNA for HBV on current or previous donation, anti-HBs (antigen to HBV antibody “s”) without vaccination against hepatitis B [17]. 

Yet another method for estimating HBV incidence with routine serologic markers in blood donor screening (HBsAg and anti-HBc), known as “HBsAg yield approach,” has been proposed and tested by various authors during the 2010 decade [16, 18–20]. A recent comparison of the most popular method based on stand-alone HBsAg [8, 9] and the HBsAg yield approach showed very close agreement of the HBV incidence estimates in the United States blood donor population [20]. 

A major advantage of the HBsAg yield approach over the stand-alone HBsAg method is to provide HBV incidence estimate for the first-time donors as well. It also makes use of both serologic markers routinely used for HBV screening in blood donor candidates. This paper extends this idea to include anti-HBc seroconverting repeat donors when the time between the last seronegative and the first seropositive test result for this marker was less than a year. Such a short period makes the assumption of a recent HBV infection more plausible. As anti-HBc marker is much more prevalent than the HBsAg marker, the precision of the estimates based on Poisson distribution of the incident cases using both markers also tends to improve. 

## 2. Material and Methods

The main objective of this paper was to compare three methods to obtain hepatitis B incidence estimate and residual risk for blood donors in southern Brazil: the stand-alone HbsAg method [8, 9], the HBsAg yield approach, and an extension to the latter which includes anti-HBc seroconverting repeat donors. In addition, deferral rate for HBV serological markers was also calculated for both repeat and first-time donors.

Computerized data from four blood banks in the state of Santa Catarina, southern Brazil, were gathered for the period 2003–2006. The blood banks were located in the cities of Florianopolis, Joinville, Criciúma, and Lages. Over 95000 blood donor candidates with complete serological test results were included in the analysis. Among these, 36350 were repeat donors during the 2003–2006 period and their total follow-up time summed to 103819 person-years. 

Serological tests in blood banks in Lages, Criciuma, and Joinville included enzyme-linked immunosorbent assays (ELISA) for HBsAg (produced by “Organon Teknika BV”) and anti-HBc (produced by “Ortho Diagnostic Systems”). In the blood bank in Florianopolis, the serological tests included microparticle enzyme immunoassay (MEIA) for HBsAg and anti-HBc (produced by “ABBOTT Laboratories”). Mean infectious window periods were 59 and 82 days for HBsAg and for the second-generation anti-HBc, respectively [15]. 

Other screening tests included EIA for HIV-1 and HIV-2, hepatitis C antibody, human T-cell lymphotropic virus type 1 and 2, antibody for *Treponema pallidum* (using Veneral Disease Research Laboratory test) and antibody for *Trypanosoma cruzi*. For the purpose of blood donation screening, positive test results for any blood screening test eliminate the possibility of transfusing the blood to a recipient. As this paper focuses on HBV transmission, the outcome of interest here is having a positive test result for either HBsAg or anti-HBc or both. 

Next section briefly describes the three methods for hepatitis B incidence estimation. For all methods, corresponding residual risk was calculated by multiplying the HBV incidence estimate by the duration of infectious window period.

### 2.1. The Stand-Alone HBsAg Method

Residual risk for HBV is the probability of seroconversion during infectious window period [8, 9]. It is calculated as the product of HBV incidence and duration of the window period. For repeat donors, un unbiased estimate of the date of seroconversion is the the midpoint of the interval between the last seronegative and subsequent seropositive test result for HBsAg. The incidence numerator is the number of HBsAg seroconverting donors, and its denominator is the time at risk for HBV infection for all blood donors tested except those who would be eliminated for some other reason (e.g., tested positive for some other serologic marker, inadequate weight, risky sexual behavior). 

However, some seroconverting donors show transient antigenemia and do not reach the level of HBsAg high enough to be detected by the test [9]. Therefore, the following adjustment is made. The probability of transient antigenaemia, denoted by *S*, is estimated by dividing its average duration of 63 days with the median interdonation period among HBsAg seroconverting donors [8]. The overall probability of detecting a new case of HBV infection, denoted by *P*, is based on the study that showed 70% prevalence of donors with transient antigenaemia and 5% prevalence of long-term HBV carriers, leading to the formula *P* = 0.7*S* + 0.05. The reciprocal value of *P* gives the adjustment factor to be multiplied by HBsAg incidence, providing corrected HBV incidence. 

### 2.2. HBsAg Yield Method

The central idea of this approach is to determine the fraction of blood donors who tested positive for HBsAg and negative for anti-HBc, separately for repeat donors and first-time donors, as well as the ratio of the two [16, 18–20]. Repeat blood donors also provide the information on the interval between the last seronegative and the first seropositive test result which can be used to estimate their exposure time and thus density-type incidence by dividing the number of seroconverting donors with corresponding person-time at risk for being an HBsAg yield case. Multiplying this estimate by the above ratio extrapolates the incidence estimate to the first-time donors as well. 

In this study, the donors with positive test results on HBV screening were verified by follow-up test results and S/O values for HBsAg. Only the donors whose S/O values equaled or exceeded the cutoff of 6 on screening and whose anti-HBC negative test results on screening was followed by subsequent anti-HBC positive test result were considered HBsAg yield cases [20]. 

### 2.3. HBsAg Yield Method with Inclusion of Anti-HBc Seroconverting Repeat Donors

Although a large multicenter study showed the lack of specificity of anti-HBc EIA tests in diagnosing acute HBV infection [21], this is less problematic in the case of repeat donors with the interdonation interval between the last two donations being less than a year. The most plausible cause of such seroconversion is an acute HBV infection. The time at risk for HBV infection and incidence are calculated in the same way as for HBsAg yield approach, only now it is extended to anti-HBc seroconverting repeat donors. In other words, this approach counts both HBsAg and anti-HBc seroconverting repeat donors in the incidence numerator. The seroconverting anti-HBc repeat donors were repeatedly tested in the followup of the screening test, and only those who repeated a positive anti-HBc test result were considered confirmed incident cases of HBV infection.

### 2.4. Statistical Methods

Descriptive statistics provide some basic information of blood donor characteristics (age, sex, number of previous donations) and HBV screening test results (HBV surface antigen and core antibody) with 95% confidence intervals based on binomial distribution. 

A recent HBV infection was considered confirmed when donor tested positive on two consecutive tests for HBsAg and suspected if the second test has not been made. The fraction of confirmed cases was multiplied by the confirmation rate among those with two consecutive tests for HBsAg to estimate the likely number of incident HBV cases in blood donors who were invited for the second HBsAg test after the first one showed a positive result but failed to do so. This number was added to the number of confirmed cases to produce the estimate of the number of seroconverting repeat blood donors with 95% confidence intervals based on Poisson distribution. 

The 95% confidence interval (CI) for the residual risk was estimated using the 95% CI for incidence, which in turn was based on the exact 95% Poisson CI for its numerator. In addition, the HBsAg yield method has the duration of the so-called yield window period (the time between HbsAg and anti-HBc positive test results) as another source of variation. As present study found only one HBsAg yield case and therefore observed no yield window variation, this information was based on simulation of a Poisson distribution (*n* = 1000) with the mean equal to the observed yield window of 45 days. Thus the 95% CI for incidence was the span between the percentiles of 2.5 and 97.5 of the simulated Poisson distribution. Stata statistical software was used for the simulation [22].

For the stand-alone HBsAg method and its extension to anti-HBC seroconverting donors, residual risk was calculated by multiplying the estimated HBV incidence by the duration of infectious window period of 59 days (0.1615 years) for the HBsAg test. In addition, a refined infectious window period of 30 and 38 days was used to make the results comparable to the seminal US study for the HBsAg yield method [20]. The span between 30 and 38 days corresponds to the time range necessary to produce 1 to 20 copies of HBV DNA per 20 mL, respectively, as a minimal infectious dose for a transfused component [23]. Both extremes of the time range were used and the results averaged to obtain the residual risk estimate for this method.

## 3. Results

Some basic characteristics of the blood donors analyzed are presented in Table 1.

Age distribution was similar for first-time and repeat donors, although the former had somewhat higher percentage of male donors and almost 20% lower percentage of donations not directed to any specific recipient such as a member of family or a friend. 

Median interdonation period for repeat donors was 507 days with interquartile range between 277 and 811 days. 

Deferral rate due to the HBV serological markers was 70 times higher in the first-time donors compared to the repeat donors (Table 2). The difference between male and female deferral rate was statistically significant for the first-time donors but not for the repeat donors. 

Based on stand-alone HBsAg, the HBV incidence in the repeat blood donors was estimated at 9.91 per 100.000 per year. The corresponding residual risk was 1 : 62482 blood donations with 95% confidence interval between 1 : 11213 and 1 : 2476780 donations (Tables 2 and 3). 

By adding ten anti-HBc seroconverting donors to the one HBsAg seroconverting donors the estimate of recent HBV seroconversions increased to 11, producing HBV incidence of 20.09 per 100.000 per year and associated residual risk of 1 : 30821 donations with 95% CI between 1 : 17229 and 1 : 61734 donations (Table 4).

Among the 116 first-time donors who tested HBsAg positive and anti-HBC negative on screening, 86 was considered confirmed positive by S/O values greater than 6 and subsequent anti-HBC positive results. Only one of three repeat donors who tested HBsAg positive and anti-HBC negative on screening were confirmed by the same criteria, as the other two donors tested subsequently negative on anti-HBC on two occasions (3 and 6 months after the screening). All ten anti-HBc soroconverting repeat donors within 12 months of their last donation tested repeatedly positive for this serological marker but none tested positive for HBsAg. None of the donors who tested positive on either HBsAg or anti-HBc on screening was found positive on anti-HBs test in the followup. 

In order to make the above results comparable to a recent US study [20], the residual risk and corresponding 95% CI for the HBsAg yield rate method were also calculated using 30 and 38 days for the infectious window period [23] and taking the average. For the repeat donors, the estimates were 1 : 41934 and 1 : 53184, with the mean of 1 : 47559 and 95% CI between 1 : 12280 and 1 : 2100046. For the first-time donors, the corresponding values were 1 : 807 and 1 : 1023 with the mean value of 1 : 915 and 95% CI between 1 : 552 and 1 : 1435. 

The ratio of the first-time to repeat donor yield rate was 51.97 (Table 5), thus indicating almost 52 times higher HBV incidence in the former. Weighted mean HBV incidence per 100000 for all donors was 751.67 with corresponding residual risk of 1 : 824. 

## 4. Discussion

HBV incidence estimates per 100000 for repeat donors were pretty close between the HBsAg yield method (20.09) and the method that included recent anti-HBc seroconverting donors (22.93) but more than twice the value obtained by the stand-alone HBsAg method (9.91). However, the difference among the estimates should be interpreted with caution as only one case of HBsAg seroconversion and relatively small sample size for this type of study may considerably reduce their precision. 

Although the confidence intervals for corresponding residual risk estimates overlapped considerably for all methods, they were extremely wide for both the HBsAg yield method and the stand-alone HBsAg method. Only the method including recent anti-HBc seroconverting donors produced reasonable confidence intervals that may be useful for comparison purposes. Smaller variance of the latter method was due to the increase in the total number of seroconverting donors by adding the anti-HBc seroconversions within the last 12 months to the HBsAg seroconversions in repeat donors. Anti-HBc seroconversion within such a short period is most likely a recent case of HBV infection as it was confirmed by repeatedly positive anti-HBc test results for all cases in the followup to the positive screening test result. It should be noticed that the stand-alone HBsAg method does not account for the variation in the adjustment factor due to the variation of the median of the interdonation period among repeat donors, neither does the HBsAg yield method account for the HBsAg yield rate variation in the population, thus underestimating the confidence intervals for residual risk. Nevertheless, in smaller settings such as regional or even smaller national blood banks, the confidence intervals can still be prohibitively high for comparison purposes. The key reason for this is the rarity of confirmed HBsAg seroconversion even in a high HBV prevalence area analyzed in the present study. The transient nature of this marker and its relatively high false positive rate when compared to anti-HBc underline the limitations of the HBV incidence methods based solely on the HBsAg test result. 

The above finding is different from that of the US study that found a close agreement between the stand-alone HBsAg and HBsAg yield rate method [20]. However, the US study also found the HBV incidence ratio of first-time to repeat donors to be 2.42 compared to almost 52 found with the data in hand. 

The importance of anti-HBc for HBV screening has been demonstrated in numerous studies. In the United States, HBV DNA testing of HBsAg negative blood showed that anti-HBc detected HBV in 1 per 49000 transfusion units that would be eligible for transfusion, which is a rate comparable to the estimated residual risk for HBV infectious window period [24]. In Asia, there is a great concern with preventing the transmission of occult HBV infection given high HBV prevalence in this part of the world. HBsAg negative blood can still contain HBV during chronic stages of infection, and anti-HBc testing can greatly increase the chance of its detection, especially in low endemic areas [25]. However, in areas with anti-HBc prevalence of 10% or higher, this strategy may not be sufficient to meet the increasing demands for blood safety, thus calling for nucleic acid amplification tests (NAT) [25]. 

Many commercially available kits for anti-HBc have demonstrated high sensitivity and reasonable specificity for high values of cut-off but only one of them has shown high specificity for moderate cut-off values among first-time donors in Germany [26]. This means that false positive test results are likely in low endemic areas such as Germany, with anti-HBc seroprevalence of 1.8% in first-time donors. However, this problem is bound to be considerably diminished for repeat donors whose anti-HBc seroconversion was registered within the last 12 months and who tested repeatedly positive, as in the present study with Brazilian blood donors. A more recent German study confirmed adequate sensitivity of most anti-HBc tests used for routine blood screening but also reported their improved specificity [27]. 

Although occult HBV infection is a great concern in Asia [25, 28, 29], it seems rare among blood recipients in low endemic areas, including in Brazil [30]. HBV is likely to be more prevalent in immunocompromised patients such as those with HIV but it is highly unlikely that both HIV and HBV would be missed on blood donor screening [31]. In addition, clinical observation indicated low transmission rate of occult HBV compared to the window period, especially in the presence of anti-HBs [32]. On the other hand, anti-HBc-positive and HBsAg negative blood donors without detectable anti-HBs are at least moderately infectious and may be highly infectious in immunocompromised blood recipients [30]. 

The magnitude of HBV incidence in southern Brazil is striking when compared to the developed countries. For example, in the United States the incidence per 100000 during the 2006–2008 period was estimated at 2.47 and 6.97 for repeat and first-time donors, respectively [20]. This compares to the present study data on the same scale in the range of 9.91 to 22.93 for repeat donors. However, it is among the first-time donors where the incidence of 1191 per 100000 in southern Brazil is more than 170 times that of the US estimate. Unlike the US study where the HBV incidence ratio from first-time to repeat donors was 2.42, in the present study it almost reached the value of 52. The deferral rate due to HBV screening serological markers of 2.13% among Brazilian first-time donors is also higher than in the Unites States. 

Other studies from the same region also found very high HBV incidence but also showed the data pointing out to considerable variability in the ratio of the first-time to repeat donor yield rate [10–12, 33]. The most recent one found this ratio close to 26 for the whole state of Santa Catarina and a considerable regional variation [33]. Such a large variation is certainly a limiting factor for precision and interpretation of HBV incidence and associated residual risk.

The main reason for such a large difference in HBV incidence between Brazil and the United States is the late start of universal vaccination of children in Brazil, so that the prevention of HBV infection is restricted to the population of less than 20 years of age, which is only a small part of the total blood donor population. Although HBV vaccination campaigns in Brazil started in the beginning of the 1990 decade, adequate vaccine supplies for universal vaccination in children were available only after 1998 [34]. In southern Brazil, the vaccine coverage currently exceeds 90% in the first year of life but less than 20% of the newborn are vaccinated within 48 hours after birth, thus missing the opportunity to reduce vertical transmission. Under the circumstances, HBV vaccination directed specifically to blood donors seems the most efficient way to bridge the gap between older unvaccinated donors and younger HBV-vaccinated generation which is yet to become eligible for blood donation.

There are several limitations of the study presented here that should be borne in mind. First, despite the population of almost 100000 donors analyzed in our study, it is still of limited size for rare outcomes such as HBsAg seroconversion in repeat donors, thus leading to a large variation of the HBV incidence and residual risk estimates. Second, HBV DNA testing was not available to obtain a more precise estimate of the HBV incidence against with which the three methods used in this work could be compared. Third, the incidence of occult HBV infection was beyond the scope of this work but is certainly an important topic to be addressed in future studies of this type.

Despite the above limitations, this is to our knowledge the first study to apply HBsAg yield method to Brazilian blood donors and compare its results with other methods, including a novel approach of counting recently seroconverted anti-HBC donors as incident cases of HBV infection. Larger population studies are needed to overcome the shortcomings of the present data. An opportunity of this kind is envisaged with the participation of some Brazilian blood banks in the REDS-II project. 

## 5. Conclusions

Although all three methods for estimating HBV incidence and residual risk compared in this study (stand-alone HBsAg, HBsAg yield method, and HBsAg yield method enhanced by anti-HBc seroconversion within the last 12 months) produced overlapping 95% confidence intervals, the last two methods produced incidence estimates about twice as high as the first one. Adding recently seroconverting anti-HBc donors to the HBsAg positive ones considerably reduced the variance of the HBV incidence and residual risk estimates. The first-time donors had about 52 times higher HBV incidence as estimated by the HBsAg yield method. As the benefits of universal child vaccination against HBV have not yet reached the blood donor population in Brazil, enhancing the HBV vaccination of blood donors remains a valuable policy.

## Figures and Tables

**Table 1:** Demographic characteristics of blood donor candidates, collection site, and intended recipient in southern Brazil, 2004–2006.

Donor profile	Categories	First-time donors		Repeat donors		All donors	
		*n*	%	*n*	%	*n*	%

Sex	Male	20001	33.90	10250	28.12	30251	31.70
	Female	38997	66.10	26195	71.88	65192	68.30

Age (years)	18-19	5805	9.84	1712	4.70	7517	7.88
	20–29	24859	42.14	15423	42.32	40282	42.21
	30–39	15322	25.97	10042	27.55	25364	26.58
	40–49	9382	15.90	6471	17.76	15853	16.61
	50–65	3630	6.15	2797	7.67	6427	6.73

Blood collection site	Blood bank	53600	90.85	34355	94.27	87955	92.15
	Mobile unit	5398	9.15	2090	5.73	7488	7.85

Donation directed to	Any recipient	33591	56.94	27731	76.09	61322	64.25
	Specific recipient	25164	42.65	8679	23.81	33843	35.46
	Self	24	0.04	14	0.04	38	0.04

**Table 2:** Deferral for either anti-HBc or HBsAg positive test result among candidates for blood donation in southern Brazil, 2004–2006.

	First-time blood donors			Repeat blood donors		
Sex	Tested	Confirmed	Prevalence (%) and CI^1^	Tested	Confirmed	Prevalence (%) and CI^1^

Female	19894	375	1.88	10215	2	0.020
			1.70–2.08			0.002–0.071
Male	38881	875	2.25	26135	9	0.034
			2.11–2.40			0.016–0.065
All	58775	1250	2.13	36350	11	0.0303
			2.01–2.25			0.015–0.054

^1^95% exact binomial confidence interval.

**Table 3:** HBV incidence and residual risk for stand-alone HBsAg in repeat blood donors in southern Brazil, 2004–2006.

Parameter	Value
Number of HBsAg seroconverting donors	1
Median of interdonation intervals (days)	329
Probability of transient antigenaemia^1^	0.1915
Probability of detecting HBV seroconversion by stand-alone HBsAg test^2^	0.1840
Time at risk (person-years) for HBsAg seroconverting donors	0.45
Time at risk (person-years) for all repeat donors	54788.73
Incidence per 100.000 per year (95% CI)^3^	1.825 (0.00, 6.73)
Adjustment factor^4^	5.43
Adjusted incidence per 100.000 per year (95% CI)	9.91 (0.00, 36.54)
Residual risk (95% CI)^5^	1 : 62482 (1 : 16946, 1 : ∞)

^1^Assuming 63 days of average duration for transient antigenaemia [8], that is, 63/329.

^2^Assuming 75% of blood donors with ELISA detectable HBsAg8.

^3^Confidence interval.

^4^Reciprocal value of the probability of detecting HBV seroconversion by stand-alone HBsAg test, that is, 1/0.184 = 5.43.

^5^Assuming Poisson distribution for one HBsAg seroconverting case.

**Table 4:** HBV incidence and residual risk for stand-alone HBsAg and anti-HBc seroconverting repeat blood donors in southern Brazil, 2004–2006.

Parameter	Value
Number of HBsAg or anti-HBc seroconverting donors^1^	11
Time at risk (person-years) for HBsAg or anti-HBc seroconverting donors	24.93
Time at risk (person-years) for all repeat donors	54788.73
Incidence per 100.000 per year (95% CI)^2^	20.09 (10.03, 35.94)
Residual risk (95% CI)^3^	1 : 3082 (1 : 17229, 1 : 61734)

^1^One HBsAg positive and anti-HBc negative, and ten HBsAg negative and anti-HBc positive donors.

^2^Confidence interval.

^3^Assuming Poisson distribution for 11 seroconverting cases.

**Table 5:** HBsAg yield rate method for first-time and repeat blood donors in southern Brazil, 2004–2006.

Parameter	First-time (*n* = 58679)	Repeat (*n* = 35432)
HBsAg− & anti-HBc−^1^	57435	35404
HBsAg− & anti-HBc+^1^	1122	10
HBsAg+ & anti-HBc−^1^	86	1
HBsAg+ & anti-HBc+^1^	6	0
Yield rate (per 100000)	146.56	2.82
Incidence^2^ per 100000 (95% CI)^3^	1191.71 (848.80, 1741.28)	22.93 (0.58, 78.30)
Residual risk (95% CI)^4^	1 : 520 (1 : 730, 1 : 356)	1 : 27003 (1 : 7908, 1 : 1067578)

^1^Number of blood donors for each HBV markers combination (“−” and “+” after the markers stand for negative and positive test results, resp.).

^2^For the repeat donors, the incidence was calculated by dividing the yield rate by the 45 days (0.123 years) of time between the anti-HBc negative test result on screening and subsequent anti-HBC positive for the one HBsAg yield case. For the first time donors, the incidence was estimated by multiplying the repeat donors incidence with the ratio of the first-time to the repeat donors yield rate.

^3^Confidence interval.

^4^Using 59 days for the infectious window period.
